# The Imbalance of FOXP3/GATA3 in Regulatory T Cells from the Peripheral Blood of Asthmatic Patients

**DOI:** 10.1155/2018/3096183

**Published:** 2018-06-14

**Authors:** Tiantian Chen, Xiaoxia Hou, Yingmeng Ni, Wei Du, Huize Han, Youchao Yu, Guochao Shi

**Affiliations:** ^1^Department of Pulmonary and Critical Care Medicine, Ruijin Hospital, Shanghai Jiao Tong University School of Medicine, Shanghai 200025, China; ^2^Institute of Respiratory Diseases, Shanghai Jiao Tong University School of Medicine, Shanghai 200025, China; ^3^Department of Pulmonary, Tongren Hospital, Shanghai Jiao Tong University School of Medicine, Shanghai 200025, China

## Abstract

**Background:**

Treg cells play an important role in the pathogenic progress of asthma.

**Objective:**

To address the alterations of Treg cells in asthma.

**Methods:**

Proliferation-and function-associated markers of Treg cells along with the percentage of Treg cells producing some cytokine from asthmatics and healthy subjects were analyzed by flow cytometry. Besides, the expressions of USP21 and PIM2 in Treg cells were measured by cell immunochemistry after Treg cells were sorted.

**Results:**

Treg cells from asthmatic patients showed lower proliferation activity and were more likely to be apoptotic. These cells expressed lower levels of GITR, CTLA-4, Nrp-1, and IL-10 compared to those from the healthy control. Th2-like Treg cells increased in asthmatic patients, while the percentage of IFN-r^+^ Treg cells was similar between two groups. Moreover, the percentage of IL-4^+^ Treg cells is related to the asthma control. Treg cells from asthmatic patients expressed more FOXP3 as well as GATA3; the expression level of GATA3 negatively correlated with FEV1%pred. Increased expressions of USP21 and PIM2 in Treg cells from asthmatic patients were found.

**Conclusion:**

Treg cells decreased in asthmatic patients, with an impaired immunosupression function and a Th2-like phenotype, which may be due to overexpression of GATA3 and FOXP3, regulated by USP21 and PIM2, respectively.

## 1. Introduction

Asthma is a heterogeneous disease of the lung and the airway characterized by chronic inflammation, airway hyperresponsiveness (AHR), and tissue remodeling [1]. The prevalence of this disease has markedly increased over the past several decades and has now become one of the major global health problems affecting approximately 300 million people worldwide [2]. Asthma pathogenesis involves multiple cell types of innate and adaptive immunity [3]. A large body of data provided evidence that activated T helper type 2 (Th2) cells played a central role through producing cytokines such as IL-4, IL-5, and IL-13 [4, 5]. Moreover, accumulating evidences of the important effect of regulatory T (Treg) cells in the mechanism of asthma have been replicated in numerous studies.

Treg cells were initially described as a population of CD4^+^T cells expressing the IL-2 receptor *α* chain (CD25) and CD45RB, able to protect mice from developing autoimmune diseases [6]. Further studies revealed that Treg cells also play an important role in other diseases, such as asthma. Mice deficient in Treg cells exhibit allergic inflammation within mucosal sites, specifically leading to pathology characteristic of asthma [7]. On the other hand, administration of galectin-9 attenuated the inflammation of *Dermatophagoides farinae*-induced chronic asthma in mice by expanding Treg cells and enhancing transforming growth factor-beta (TGF-*β*) signaling [8]. Our previous study showed that the percentage of Treg cells was significantly lower in the peripheral blood of patients with moderate to severe allergic asthma than in patients with mild asthma or the control group [4]. However, further studies are needed to investigate the other alterations of Treg cell in patients with asthma besides cell number.

Forkhead box P3 (FOXP3), the specific transcription factor, plays a necessary and sufficient role in the development and function of Treg cells [9]. Other transcription factors were needed to coordinate with Foxp3 to weaken the immunological effect of effector T cells [10]. Among these transcription factors, GATA3 is crucial for the function of Treg cells in limiting Th2-type inflammatory responses, which indicates that GATA3 in Treg cells may be relevant to the pathogen of asthma.

GATA3 and FOXP3 can be regulated by different mechanisms. In the terms of GATA3, E3 deubiquitinase ubiquitin-specific peptidase 21 (USP21) can upregulate the expression of GATA3 in Treg cells [11]. USP21 belongs to the deubiquitinase family, which opposes the function of E3 ubiquitin ligases [12]. In previous studies, Zhang has revealed that USP21 interacted with GATA3 to promote its stability via deubiquitination and the knockdown of USP21 resulted in the downregulation of GATA3 protein levels in Treg cells [11]. What is more, in the recent published article, it was revealed that PIM2, a kind of serine/threonine kinases, could phosphorylate the Foxp3 N-terminal domain, thus negatively regulating Treg cell suppressive function by influencing the Foxp3 level and expression of Treg cell-associated surface markers [13]. Most importantly, the mRNA levels of USP21 and PIM2 were upregulated in the Treg cells of asthma patients [11]_._ So we hypothesized that there might be a change in the expressions of USP21 and PIM2 of Treg cells.

## 2. Methods

### 2.1. Subjects

The population consisted of patients with asthma aged between 16 and 65 years from Ruijin Hospital (Shanghai, China). The diagnosis of asthma was based on the GINA guidelines. Subjects had received a physical examination, spirometry, and asthma control assessment (ACQ-7 questionnaire). According to the GINA guideline, a score of 0.0–0.75was classified as well-controlled asthma and >0.75 as partly/poorly uncontrolled asthma. Subjects were excluded if they had experienced an asthma exacerbation in the previous four weeks or a respiratory infection in the previous a week. All subjects gave their written informed consent before participation, and this study was approved by the ethics committee of the hospital.

### 2.2. Reagents

The culture medium used was X-VIVO media (Lonza, USA) supplemented with 10% human AB serum, 1% GlutaMAX (Invitrogen, USA), 1% sodium pyruvate (Invitrogen, USA), and 1% penicillin/streptomycin (Invitrogen, USA). Fluorochrome-conjugated anti-CD4 was from BioLegend (USA). Fluorochrome-conjugated anti-Ki67, CTLA4, and GITR were from eBioscience (USA). Fluorochrome-conjugated anti-CD45RA, TGF-*β*, IL-10, IFN-*γ*, IL-4, IL-5, IL-13, FOXP3, and GATA3 were from BD (USA). PE Annexin V Apoptosis Detection Kit I, Cytofix/Cytoperm Kit, Pharmingen™ Leukocyte Activation Cocktail with BD GolgiPlug™, and Leukocyte Activation Cocktail with BD GolgiPlug were also from BD (USA). Anti-Human CD25 PerCP-Cyanine5.5 was from eBioscience (USA), and anti-Human CD25-PE was from BD (USA). Human Neuropilin-1 PerCP MAb was from R&D (USA). Anti-Human USP21 was purchased from Sigma-Aldrich Co. (USA), and anti-Human PIM2 was from Santa Cruz (USA); the secondary antibodies were from Sigma-Aldrich Co. (USA). Recombinant human cytokine IL-2 was purchased from R&D, and rIL-4 and TGF-*β* were from PeproTech (USA). Ficoll-Paque PLUS was purchased from GE Healthcare (UK). Human IL-10 ELISA Kit and Human TGF-*β*1 ELISA Kit were from RayBiotech (USA). Anti-CD3/CD8 Dynabeads were purchased from Invitrogen (USA).

### 2.3. Peripheral Blood Mononuclear Cell (PBMC) Isolation

Sixteen milliliters of peripheral blood was obtained in a sodium heparin vacuum tube. PBMCs were isolated by Ficoll-Paque PLUS according to the manufacturer's instruction; the cells isolated were divided into several shares for the flow cytometry analysis.

### 2.4. Flow Cytometry Analysis

Treg cells were identified as anti-CD4-positive and anti-CD25-positive. Where indicated, additional markers were evaluated using anti-Human Ki-67 PerCP-eFluor® 710, human Neuropilin-1 PerCP MAb, anti-Human CD152 (CTLA-4) PE, HU FOXP3 APC, and Gata3 PE. Before intracellular staining, Cytofix/Cytoperm Kit and Pharmingen Leukocyte Activation Cocktail with BD GolgiPlug were used for cell fixation and permeation.

PE Annexin V Apoptosis Detection Kit I was used to identify the apoptosis of Treg cells. For intracellular cytokine production, PBMCs were stimulated with Leukocyte Activation Cocktail with BD GolgiPlug for 3 hours before staining. Cytofix/Cytoperm Kit and Pharmingen Leukocyte Activation Cocktail with BD GolgiPlug were used for intracellular cytokine production. Intracellular staining was performed using APC Rat anti-Human IL-4, PE Rat anti-Human IL-5, APC Rat anti-Human IL-13, PE Mouse anti-Human IFN-*γ*, APC Rat anti-Human IL-10, and PE Mouse anti-Human TGF-*β*1.

Samples were detected with a FACS flow cytometer, and acquired data were analyzed with FlowJo software.

### 2.5. Treg Cells and Naïve T Cell Isolation

Fifty milliliters of peripheral blood was obtained in a sodium heparin vacuum tube, and PBMCs were isolated according to the above mentioned. PBMCs were stained with FITC anti-human CD4 antibody, anti-CD25-PE, and anti-CD45RA-APC at 4°C for 30 minutes in the dark, then CD4^+^CD25^+^CD45RA^−^Treg and CD4^+^CD25^−^CD45RA^+^ naïve T cells were isolated from PBMC by a BD FACSAria II sorter. CD4^+^CD25^+^CD45RA^−^Treg cells were prepared for the analysis of the expressions of USP21 and PIM2. CD4^+^CD25^−^CD45RA^+^ naïve T cells were cultured in vitro for the analysis of the ability to develop Treg cells.

### 2.6. The Expression of USP21 and PIM2 in Treg Cells

Freshly sorted CD4^+^CD25^+^CD45RA^−^Treg cells were swung to the slide by StatSpin CytoFuge 12. Then, the cells in the slides were fixed in 4% paraformaldehyde for 10 minutes and permeabilized with 1% Triton-100. After being washed with PBS, samples were incubated with anti-Human USP21 or anti-Human PIM2 in 4°C overnight. After three washings with PBS, cells were incubated with secondary antibodies for 2 hours at room temperature. Then, USP21 or PIM2 was stained with DAB Color Developing Reagent Kit, and nuclei were stained with hematoxylin. These samples were examined on a Nikon Eclipse 50i microscope (Nikon, Japan), and the images were analyzed with Image-Pro Plus 6.0.

### 2.7. TGF-Beta-Mediated In Vitro Treg Cell Induction

Sorted CD4^+^CD25^−^CD45RA^+^ naïve T cells were cultured with anti-CD3/CD8 Dynabeads at a cell-to-bead ratio of 1 : 3 in X-VIVO media supplemented with 10% human AB serum, 1% GlutaMAX, 1% sodium pyruvate, 1% penicillin/streptomycin, recombinant TGF-*β* (5 ng/ml), retinoic acid (10 nM), and a gradient of recombinant IL-4 (0, 0.625 ng/ml, 1.25 ng/ml, 2.5 ng/ml, 5 ng/ml, and 10 ng/ml, resp.). After 3 days, the CD4^+^CD25^+^ Treg cells were analyzed by flow cytometry.

### 2.8. Statistical Analysis

Data were graphed and analyzed by Prism 6 software (GraphPad). Statistical significance between two groups was determined by two-tailed Student's *t*-test. Pearson test was used for the analysis of correlation. *P* values less than 0.05 was considered statistically significant.

## 3. Results

### 3.1. Deficiency of Treg Cells in Patients with Asthma

**Figure 1:**
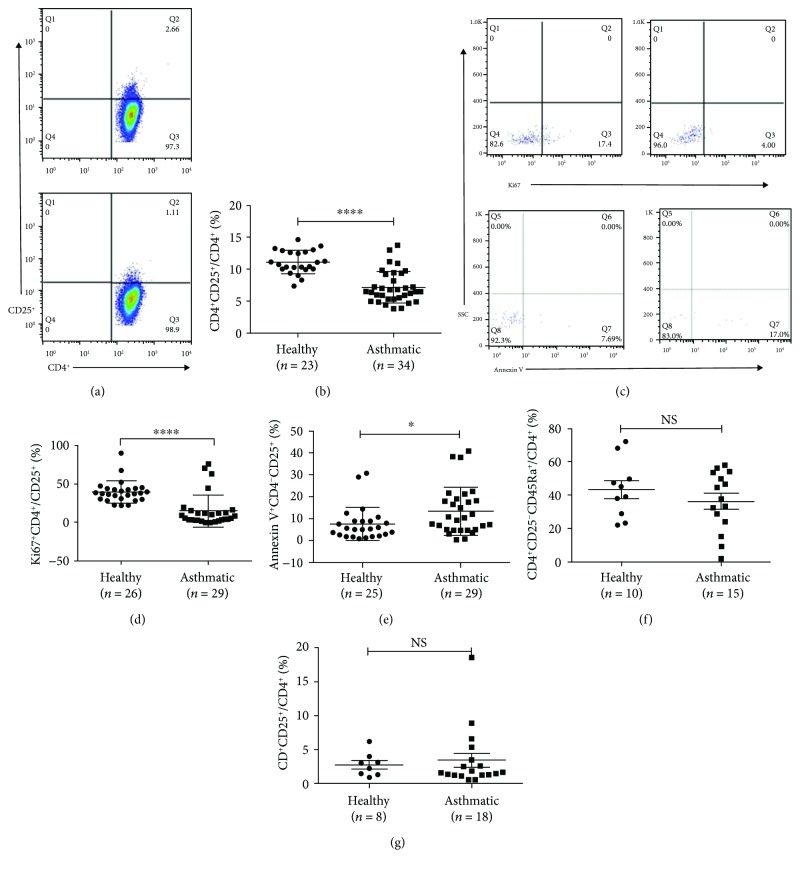
Deficiency of Treg cells in patients with asthma. (a) Representative examples of flow cytometry analysis of CD4^+^CD25^+^ Treg cells in healthy and asthmatic subjects. (b) Frequencies of CD4^+^CD25^+^ Treg cells in CD4^+^ T cell subsets in healthy and asthmatic groups. (c) Representative examples of flow cytometry analysis of Ki67^+^ and Annexin V^+^ cells of CD4^+^CD25^+^ Treg cells in healthy and asthmatic subjects. (d) Frequencies of Ki67 and Annexin V (e) production in healthy and asthmatic subjects. (f) The percentage of CD4^+^CD25^−^CD45RA^+^ T cells of CD4^+^ T cell subsets in healthy and asthmatic groups. (g) Percentage of Treg cells induced from naïve T cells of healthy and asthmatic groups in Treg cell polarity. The graph shows means ± sem. ^∗^*p* < 0.05 and ^∗∗∗∗^*p* < 0.0001. NS: no significance.

Consistent with the previous studies, the percentage of Treg cells in the asthmatic group was lower than that in healthy group ((7.24 ± 2.49)% versus (11.13 ± 1.82)%, *p* < 0.01, Figures 1(a) and 1(b)). To further investigate the reason of the decreasing number of Treg cells, we analyzed the expression of Ki67 in Treg cells, and we found that Treg cells from the asthmatic group expressed less Ki67 than those from the healthy group ((15.04 ± 20.91)% versus (39.78 ± 14.22)%, *p* < 0.05, Figures 1(c) and 1(d)), which meant decreased proliferation of these cells. The same samples also showed evidence of increased apoptosis in the asthmatic group compared to healthy ones ((13.52 ± 11.0)% versus (7.62 ± 7.63)%, *p* < 0.05), as revealed by Annexin V staining (Figures 1(c) and 1(e)). In vitro, differentiation of CD4^+^CD25^−^CD45RA^+^ naïve T cells into Treg cells was the same in both groups (healthy versus asthmatic (43.54 ± 17.11)% versus (36.60 ± 17.82)%, *p* > 0.05, Figure 1(f)). The culture of naïve T cells in Treg cell polarity showed that these cells in two groups had the same ability to differentiate into CD4^+^CD25^+^ Treg cells (healthy versus asthmatic (2.78 ± 1.72)% versus (3.46 ± 4.37)%, *p* > 0.05, Figure 1(g)).

### 3.2. Defective Function of Treg Cells in Immunosuppression

**Figure 2:**
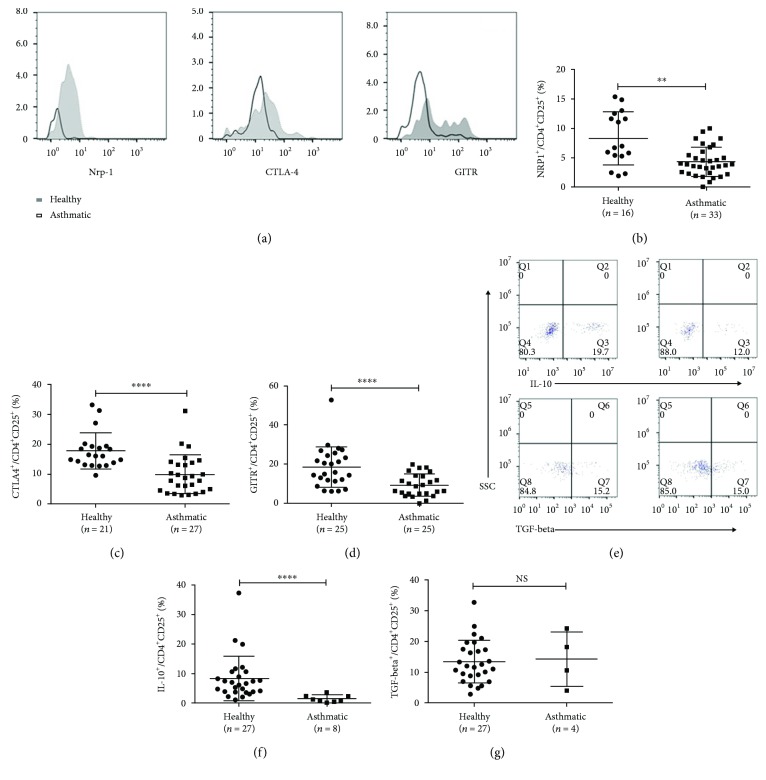
Defective function of Treg cells in immune-suppression. (a) Representative examples of flow cytometry analysis of Nrp-1, CTLA-4, and GITR expression by CD4^+^CD25^+^ Treg cells in healthy and asthmatic subjects. (b, c, d) Frequencies of Nrp-1 CTLA-4 and GITR expressed in healthy and asthmatic subjects. (e) Representative examples of flow cytometry analysis of IL-10 and TGF-beta production by CD4^+^CD25^+^ Treg cells in healthy and asthmatic subjects. (f, g) Frequencies of IL-10 and TGF-beta production in healthy and asthmatic subjects. The graph shows means ± sem. ^∗∗^*p* < 0.01 and ^∗∗∗∗^*p* < 0.0001. NS: no significance.

To establish whether Treg cells from patients with asthma are functionally competent, we investigated the expressions of Neuropilin-1 (Nrp-1), cytotoxic T lymphocyte-associated antigen-4 (CTLA-4), and glucocorticoid-induced tumor necrosis factor receptor (GITR), which are related to the suppressive function of Treg cells. And results showed that all those markers were expressed in a lower level in the asthmatic group than those in the healthy group (healthy versus asthmatic group (8.30 ± 4.53)% versus (4.31 ± 2.49)%, *p* < 0.01; (17.81 ± 6.06)% versus (9.89 ± 6.52)%, *p* < 0.01; and (18.55 ± 10.38)% versus (9.20 ± 5.70)%, *p* < 0.01, respectively, Figures 2(a)–2(d)). Moreover, in agreement with the decreased level of IL-10 in the plasma (the data not shown), the percentage of IL-10^+^ Treg cells was decreased (healthy versus asthmatic group (8.28 ± 7.56)% versus (1.41 ± 1.20)%, *p* < 0.01, Figure 2(e)), while the TGF-*β*^+^ Treg cell percentage was similar between two groups ((13.46 ± 6.92)% versus (14.31 ± 8.80)%, *p* > 0.05, Figure 2(f)).

### 3.3. Increased Number of Producing Th2-Cytokine Treg Cells

**Figure 3:**
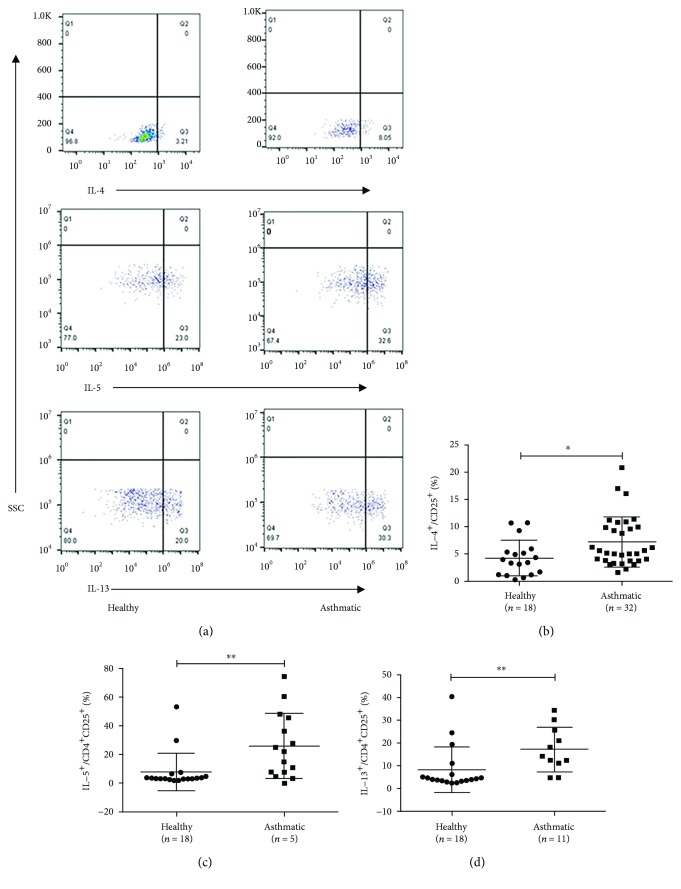
Increasing number of producing Th2-cytokine Treg cells. (a) Representative examples of flow cytometry analysis of IL-4, IL-5, and IL-13 production by CD4^+^CD25^+^ Treg cells in healthy and asthmatic subjects. (b, c, d) Frequencies of IL-4, IL-5, and IL-13 production in healthy and asthmatic subjects. The graph shows means ± sem. ^∗^*p* < 0.05 and ^∗∗^*p* < 0.01. NS: no significance.

**Figure 4:**
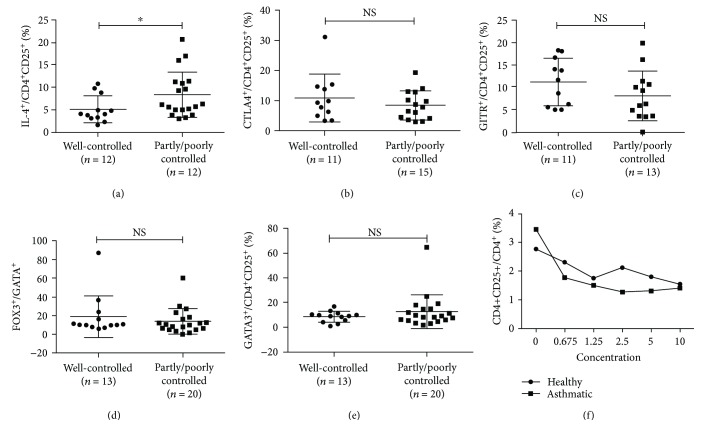
The expression of various markers in Treg cells in well-controlled and partly/poorly controlled groups. (a, c, d, e, f) Frequencies of IL-4, CTLA-4, GITR, FOXP3, and GATA3 expressing in well-controlled and partly/poorly controlled groups. (b) Effect of different IL-4 concentrations on TGF-beta induction of Treg cells from naïve T cells of healthy (*n* = 8) and asthmatic (*n* = 18) groups. The graph shows means ± sem. ^∗^*p* < 0.05. NS: no significance.

Some researchers have found that Treg cells are unstable in vivo, and these unstable Treg cells play roles in the pathogenesis of these diseases [14]. For this reason, the expressions of Th2 cytokines, IL-4, IL-5, and IL-13, and Th1 cytokine IFN-*γ* were analyzed. As shown in Figure 3, the fraction of IL-4^+^, IL-5^+^, and IL-13^+^ Treg cells increased significantly in the asthmatic group (healthy versus asthmatic group, (4.25 ± 3.26)% versus (7.20 ± 4.58)%, *p* < 0.05; (7.77 ± 13.00) versus (25.92 ± 22.72), *p* < 0.01; and (8.323 ± 10.04)% versus (17.14 ± 9.81)%, *p* < 0.01, respectively,), but no difference in IFN-*γ*^+^ Treg cells between two groups (healthy versus asthmatic group, (4.29 ± 2.71)% versus (4.02 ± 3.98)%, *p* > 0.05) (Figure 4). According to patients' clinical symptoms and lung function, they were divided into two groups, the well-controlled and partly/poorly controlled group. We found that Treg cells from partly/poorly controlled asthma patients expressed more IL-4 than those from well-controlled asthma patients ((8.36 ± 5.09)% versus (5.12 ± 3.01)%, *p* < 0.05, Figure 4(a)). However, Treg cells from partly/poorly controlled asthma patients expressed similar levels of CTLA4, GITR, FOXP3, and GATA3, compared to those from well-controlled asthma patients (Figures 4(b)–4(e)). To clarify the role of IL-4 in the development of Treg cells from naïve T cells, we cultured naïve T cells with the concentration gradient of IL-4 and found that IL-4 failed to prevent naive CD4^+^ T cell differentiation into Treg cells (Figure 4).

### 3.4. Enhanced Expression of Specific Transcription Factors, Especially GATA3 in Treg Cells from Asthmatic Patients

**Figure 5:**
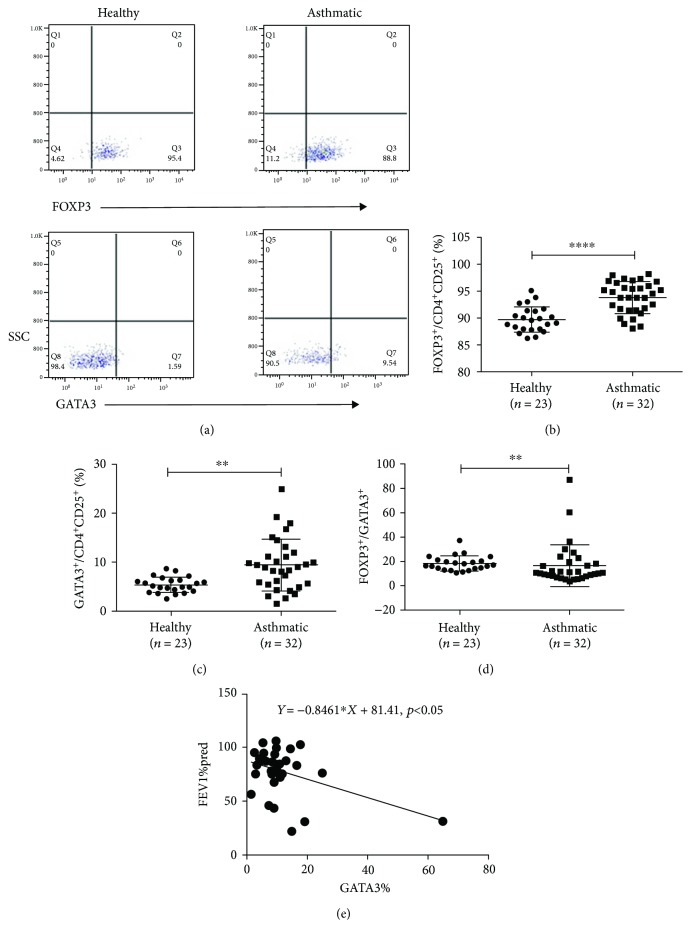
Enhancing expression of specific transcription factors, especially GATA3 in Treg cells from asthmatic patients. (a) Representative examples of flow cytometry analysis of FOXP3 and GATA3 expression by CD4^+^CD25^+^ Treg cells in healthy and asthmatic subjects. (b, c). Frequencies of FOXP3 and GATA3 expressed in healthy and asthmatic subjects. (d) The ratio of FOXP3 and GATA3 expressed in healthy and asthmatic subjects. (e) The correlation of the percentage of CD4^+^CD25^+^GATA3^+^ Treg cells with FEV1%pred. The graph shows means ± sem. ^∗^*p* < 0.05, ^∗∗^*p* < 0.01, and ^∗∗∗∗^*p* < 0.0001. NS: no significance.

In that some specific transcription factors play a decisional role in the development and maintenance of T cells, we checked the expression level of FOXP3 and GATA3 in Treg cells. Results showed that the expressions of FOXP3 (healthy versus asthmatic group, (89.81 ± 2.36)% versus (93.82 ± 2.93)%, *p* < 0.01, Figures 5(a) and 5(b)) and GATA3 (healthy versus asthmatic group, (5.37 ± 1.59)% versus (9.40 ± 5.31)%, *p* < 0.01, Figures 5(a), 5(c)) in Treg cells were increased in the asthmatic group. Interestingly, the increase in GATA3 expression was more obvious than in Foxp3 expression, leading to a decreased FOXP3/GATA3 ratio in the asthmatic group compared to the healthy group ((16.81 ± 17.13)% versus (18.34 ± 6.23)%, *p* < 0.01, Figure 5(d)). And we also found that FEV1%pred of patients with asthma was correlated with the percentage of GATA3^+^ Treg cells (Figure 5(e)).

### 3.5. Increased Expression of USP21 and PIM2 in Treg Cells from Asthmatic Patients

**Figure 6:**
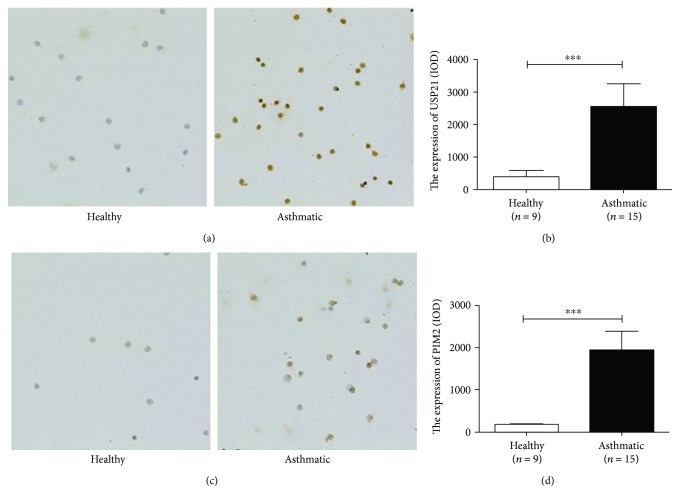
Increasing expression of USP21 and PIM2 in Treg cells from asthmatic patients. (a, c) USP21 and PIM2 staining in CD4^+^CD25^+^ Treg cells (revealed in brown, 200x). (b, d) The expression level of USP21 and PIM2 in CD4^+^CD25^+^ Treg cells from healthy (*n* = 9) and asthmatic (*n* = 15) subjects. The graph shows means ± sem. ^∗∗∗^*p* < 0.001. NS: no significance.

Previous studies showed that both USP21 and PIM2 are regulators of GATA3 and FOXP3 expression, respectively, in Treg cells, so we measured the expressions of USP21 and PIM2 in Treg cells from patients with asthma and healthy subjects. Cell immunochemistry showed that Treg cells in the asthmatic group expressed more USP21 and PIM2 than Treg did cells in the healthy group (healthy versus asthmatic group, IOD value, (390.2 ± 586.2) versus (2557 ± 2698), *p* < 0.01; (183.5 ± 106.3) versus (1935 ± 1775), *p* > 0.01, respectively, Figure 6).

## 4. Discussion

We investigated the alterations of Treg cells in the peripheral blood from patients with asthma. Multiple studies have verified that asthma is a complicated process characterized by type 2 inflammatory reactions involving the coordination of innate and adaptive immune responses [15]. Treg cells can maintain the balance of immune self-tolerance and homeostasis via limiting aberrant or excessive inflammation [16, 17], and they can regulate the effector function of all T helper cells [18], including Th2 cells, which play a central role in the pathogenic development of asthma. Our previous study has demonstrated that there is an increased ratio of Th2/Treg cells in patients with moderate to severe asthma, which suggests that Th2/Treg imbalance has an important role in asthma [4]; however, the alteration of the function besides the number of Treg cells in asthma patients needs to be further investigated.

In this study, we found that the percentage of Treg cells from asthmatic patients markedly decreased accompanied by decreased proliferation and increased apoptosis, which gave us an appropriate explanation of the decreased percentage of Treg cells in the asthmatic group. What is more, the same percentage of CD4^+^ naïve T cells and Treg cells induced in the Treg polarity indicated that there was no difference in the differentiation of naïve T cells between asthmatic and healthy subjects. Therefore, to a great extent, the decreasing percentage of Treg cell in the patients with asthma may result from the decreased proliferation and increased apoptosis.

The change takes place not only in the number of Treg cells in asthmatic patients but also in their functions. Shevach concluded that Treg cells could secrete suppressor cytokines (IL-10 and TGF-*β*) that can directly inhibit the function of responder T cells and myeloid cells [19]. In addition, CTLA-4 on Treg cells can downregulate or prevent the upregulation of CD80 and CD86, the major costimulatory molecules on antigen-presenting cells [19]. Meanwhile, Nrp-1 can promote long interactions between Treg cells and immature DCs and restrict access of the effector cells to antigen-presenting cells so as to suppress the proliferation mediated by Treg cells when the responder T cells are stimulated with low concentrations of antigen [19]. All those molecules are of great importance in the function of Treg cells, so the expressions of these markers were analyzed to evaluate the function of Treg cells, and we found that Treg cells from patients with asthma expressed less IL-10, Nrp1, and CTLA-4, which indicated the defective function of Treg cells in these patients. GITR (TNFRSF18/CD357/AITR) is a cell surface receptor constitutively expressed at high levels on Treg cells and at low levels on naïve and memory T cells [20]. Previous studies have proved that GITR is a crucial player in Treg differentiation and explanation [21]. While high GITR expression is clearly a marker for Treg cells, GITR has also been demonstrated to lead to FOXP3 loss, inhibit the expansion and suppressive activity of Treg cells, and promote Teff resistance to Treg suppression [20, 22, 23], which means excess expression of GITR may be a clue of impaired function of Treg cells. In a recent study, GITR single-positive cells (GITRsp, CD4^+^CD25^low/−^Foxp3^low/−^GITR^+^) have been found that can express high levels of CTLA4, produce much more IL-10, and have regulatory activity, meaning that GITRsp cells might play a role in decreasing T cell activation/proliferation and controlling autoimmune disease [24]. In our study, we found that Treg cells in the asthmatic group expressed less GITR than those in healthy group. This decreased level of GITR expression may not be explained by exiting theories. Herein, more experiments will be needed to investigate the role of GITR in the pathogen of asthma.

Since changes have taken place in the number and function of Treg cells in asthmatic subjects, the underlying mechanism needed to be verified next. FOXP3, an X-linked transcription factor, is highly and specifically expressed in Treg cells [25]. As the specific transcriptional factor of Treg cells, FOXP3 is the absolute need in the development and function of Treg cells [9, 26]. Besides, other transcriptional factors are also needed for the immune-suppressive function of Treg cells [10]. Among them, GATA3 plays an indispensible role in Treg cell function. Previous study has revealed that the low expression of Foxp3 seems to account for a degree of GATA3 upregulation by some mechanism that favors nTreg-to-Th2 conversion [27]; what is more, after depleting GATA3 in Treg cells of mice, these Treg cells expressed reduced amounts of Foxp3 and were enhanced in the ability to produce inflammatory cytokines, which contributed to the inflammatory disorder in mice [26]. Wang revealed that GATA3 can bind to CNS2 of the *foxp3 locus* and deletion of GATA3 specifically in Treg cells resulted in an inflammatory syndrome in mice that could be ascribed to defective function of Treg cells [25]. In addition, GATA3-deficient Treg cells expressed reduced amounts of Foxp3 [25]. On the contrary, the upregulation of GATA3 in Treg cells led to the secretion of IL-4 in Treg cells, even the conversion of Treg cells to Th2 cells [28]. In 2012, Rudra et al. have revealed that Foxp3 interacts with GATA3 in Treg cells by biochemical and mass-spectrometric analysis; meanwhile, they also verified that Foxp3 and GATA3 reciprocally increased the expression of each other at least in part through direct binding to the corresponding genetic loci [27]. Based on these experiments, we hypothesized that it was the balance of FOXP3 and GATA3 in Treg cells that could ensure its exerting immuno-suppressive effects. Therefore, we analyzed the expression of FOXP3 and GATA3 from healthy and asthmatic subjects, and we found that both FOXP3 and GATA3 expressed in a higher level in the asthmatic group; however, Treg cells expressed more GATA3 in the asthmatic group, because the ratio of FOXP3 and GATA3 decreased in this group. In other words, there was an imbalance of FOXP3 and GATA3 in Treg cells from asthmatic subjects.

As to the importance of FOXP3 and GATA3 in Treg cells, the regulation of them was focused on in our experiments. Preciously, our collaborating laboratory has verified that USP21 can colocalize and interact with GATA3 and the role of USP21 on GATA3 in FOXP3-expressing cells is certified. Overexpression of USP21 can rescue GATA3 from its degradation so as to stabilize the expression of GATA3 [11]; RT-PCR showed that the mRNA of USP21 is upregulated in the Treg cells of asthma patients. In our experiment, the increasing level of USP21 in Treg cells was shown, which is consistent with the upregulation of GATA3 in Treg cells. Deng's study proved that PIM2 is highly expressed in human Treg cells and phosphorylates FOXP3 in vitro and vivo; beyond that, knockout of PIM2 in vivo enhanced Treg cell suppressive function and stability through altered expression of FOXP3 [13]. Our previous experiment also proved that PIM2 was essential for airway inflammation and airway hyperreactivity [29]. In accordance with the above research, we found the up-expression of PIM2 and the impaired function of Treg cells in asthmatic patients. The correlation analysis showed there was no correlation between GATA3 and USP21 or FOXP3 and PIM2 (the data was not shown). We thought the explanation was that USP21 or PIM2 may regulate the expression in a complicated way rather than in a linear relationship and it is possible that other molecules participate in the regulation of Treg cells in the pathogen of asthma.

As the expression of FOXP3 can suppress the expression of downstream genes, such as Il-4, Il-5, and Il-13, on the other hand, the increased GATA3 can also promote the secretion of Th2-type cytokines [9]. We testified if the change of transcription factors leads to the change of the expression of cytokines downstream. In our experiment, Treg cells isolated from patients with asthma expressed higher levels of Th2-type cytokines, such as IL-4, IL-5, and IL-13, rather than IFN-*γ*, the Th1-type cytokine. The increased expression of GATA3 and decreased FOXP3 relatively give us a hint for the higher level of Th2 cells. On the other hand, our experiment also proved that Treg cells were unstable and it may be pathogenic in asthmatic patients. However, the ability of IL-4-, IL-5-, or IL-13-producing Treg cells in the development of asthma needs to be further investigated.

To explore the differences among asthmatic patients in different conditions, these subjects were divided into the well-controlled group and partly/uncontrolled group according to the ACQ-7 questionnaire, and it was found that IL-4 producing Treg cells were increased in the partly/poorly controlled group rather than in the well-controlled group, which means IL-4 may play a direct role in the development of asthma and affect the treatment effect of asthma. Since the percentage of Treg cells was significantly lower in the peripheral blood of patients with moderate to severe asthma than in patients with mild asthma [4], we designed different concentrations of IL-4 to imitate the different stages of asthma and to explore if IL-4 could affect the differentiation of Treg cells. We cultured naïve T cells in the culture polarity of Treg cell plus the concentration gradient of IL-4, and there was no significant difference of Treg differentiation in the different concentration of IL-4. In consideration of the important role of pathogenic Treg cells, how IL-4 produced by Treg cells individually can affect the pathogen of asthma needs more experiments. Besides, there were no significant differences of the expression of marker such as CTLA-4, GITR, FOXP3, and GATA3. It may give us the explanation that asthma is a complicated and programming pathology so that it is unreasonable to divide them explicitly.

In summary, multifaceted changes of Treg cells have taken place in the process of asthma. The imbalance of FOXP3 and GATA3 placed an important role in the function of Treg cells, which thus led to the pathogenic alteration of Treg cells, releasing more Th2-type cytokines, such as IL-4, IL-5, and IL-13. USP21 and PIM2 may exert an important role in the process by regulating GATA3 of Treg cells. However, more evidences need to be presented for the role of USP21 and PIM2 in the regulation of GATA3 and FOXP3.

## Data Availability

The data used to support the findings of this study are available from the corresponding author upon request.
